# *Plasmodium falciparum* Antigens on the Surface of the Gametocyte-Infected Erythrocyte

**DOI:** 10.1371/journal.pone.0002280

**Published:** 2008-05-28

**Authors:** Maha Saeed, Will Roeffen, Neal Alexander, Christopher J. Drakeley, Geoffrey A. T. Targett, Colin J. Sutherland

**Affiliations:** 1 Department of Infectious and Tropical Diseases, London School of Hygiene and Tropical Medicine, London, United Kingdom; 2 Radboud University Nijmegen Medical Centre, Nijmegen, The Netherlands; Federal University of Säo Paulo, Brazil

## Abstract

**Background:**

The asexual blood stages of the human malaria parasite *Plasmodium falciparum* produce highly immunogenic polymorphic antigens that are expressed on the surface of the host cell. In contrast, few studies have examined the surface of the gametocyte-infected erythrocyte.

**Methodology/Principal Findings:**

We used flow cytometry to detect antibodies recognising the surface of live cultured erythrocytes infected with gametocytes of *P. falciparum* strain 3D7 in the plasma of 200 Gambian children. The majority of children had been identified as carrying gametocytes after treatment for malaria, and each donated blood for mosquito-feeding experiments. None of the plasma recognised the surface of erythrocytes infected with developmental stages of gametocytes (I–IV), but 66 of 194 (34.0%) plasma contained IgG that recognised the surface of erythrocytes infected with mature (stage V) gametocytes. Thirty-four (17.0%) of 200 plasma tested recognised erythrocytes infected with trophozoites and schizonts, but there was no association with recognition of the surface of gametocyte-infected erythrocytes (odds ratio 1.08, 95% C.I. 0.434–2.57; P = 0.851). Plasma antibodies with the ability to recognise gametocyte surface antigens (GSA) were associated with the presence of antibodies that recognise the gamete antigen Pfs 230, but not Pfs48/45. Antibodies recognising GSA were associated with donors having lower gametocyte densities 4 weeks after antimalarial treatment.

**Conclusions/Significance:**

We provide evidence that GSA are distinct from antigens detected on the surface of asexual 3D7 parasites. Our findings suggest a novel strategy for the development of transmission-blocking vaccines.

## Introduction

Available evidence suggests that there are specific immune responses to different stages of the malaria parasite life cycle. Natural human immune responses to malaria recognise extracellular sporozoites and merozoites, which both have surface-exposed antigens, and are the targets of various vaccines currently under development [1]. Blood-stage immunity also involves the acquisition of a repertoire of antibodies (IgG) directed against parasite-encoded variant surface antigens (VSA) on the surface of the infected erythrocyte [2], [3]. Carriage of IgG which recognise VSA, including *P. falciparum* erythrocyte membrane protein-1 (PfEMP-1) [4], [5], is associated with protection from clinical malaria [6]–[11].

Transmissible sexual stages of the malaria parasite, gametocytes, frequently die in the host without being passed on to a mosquito, and in doing so release intracellular antigens into the host circulation. Among these antigens are a number that elicit humoral responses which mediate transmission blocking immunity. This occurs when human antibodies, taken up by a mosquito in a potentially infective blood-meal containing male and female *Plasmodium* gametocytes, are able to block further parasite development and prevent infection of the mosquito. This immunity is known to be antibody-mediated [12] and is directed against the parasites in the mid-gut of the mosquitoes immediately after ingestion of a blood meal by the mosquito [13]–[17]. Targets of this immunity include the gamete surface proteins Pfs230 and Pfs48/45, but other antigens may be involved. These gamete proteins are not present on the surface of intact gametocytes and thus antibodies against these antigens are unlikley to have any effect on the parasite in the human host.

By comparison, little is known about any specific immune responses that may recognise the surface of erythrocytes infected with sexual stages of malaria parasites during their development in the human body. We know that erythrocytes infected with early forms of *P. falciparum* gametocytes sequester away from the peripheral circulation until they reach maturity [18], which suggests the presence of adhesins on the gametocyte-infected erythrocyte surface. Analysis of the adhesion phenotype of stage I–V gametocytes that mediates binding to C32 melanoma cells [19] and transformed human bone marrow endothelial cells trHBMEC [20] suggests adhesion of sexual stages has some characteristics in common with, and others that differ from, asexual parasite adhesion. Further, the evidence that asexual and sexual stage parasites sequester in different tissues [20], [21] suggests that distinct antigens exist on the surface of gametocyte-infected erythrocytes (gametocyte surface antigens, GSA). Such adhesins could conceivably be either parasite-encoded molecules, altered host membrane components, or both. We reasoned that such antigens may elicit specific immune responses, independent of responses to asexual parasites, which may be capable of suppressing or killing gametocytes. No studies to date have demonstrated the presence of GSA. If the surface of gametocyte-infected erythrocytes do elicit immune responses, then a comparison of these to responses elicited by asexual parasites and to transmission-blocking antibodies would be of great interest.

We present the results of experiments in which plasma collected from 200 Gambian children carrying microscopically confirmed gametocytes 7 to 14 days after treatment for uncomplicated *P. falciparum* malaria, were presented with live, cultured *P. falciparum* gametocytes. The degree of recognition of each test plasma was measured by flow cytometry. We demonstrate for the first time the existence of antibodies which specifically recognise the surface of gametocyte-infected erythrocytes, and explore associations with recognition of asexual parasites and gamete antigens Pfs 48/45 and Pfs 230.

## Methods and Materials

### Patients and plasma samples

During the months of October to December 2000, 2001 & 2002, children under 10 years presenting as outpatients to Farafenni hospital, The Gambia, were recruited into clinical trials to study the effects of antimalarial treatment on *P. falciparum* transmission (N = 536, 500 and 497 in 2000, 2001 and 2002 respectively; refs 22–25). All participants or their parents/guardians gave informed consent to participation in these studies as previously described. Children were treated with chloroquine (CQ), sulphadoxine-pyrimethamine (SP), CQ/SP in combination, CQ combined with artesunate (CQ/AS) or artemether-lumefantrine (AL).

Seventy, 72 and 27 children in each year respectively were identified as gametocyte carriers 7 days after treatment and donated venous blood for membrane-feeding of *Anopheles* mosquitoes as previously described [23]–[26]. In 2002, an additional 35 patients, randomly selected from among those without gametocytes 7 days after treatment, donated blood for membrane-feeding of mosquitoes, giving a total of 204 experiments. Each blood sample was collected in the anti-coagulant citrate phosphate dextrose, and the plasma separated from cells by centrifugation. 200 plasma samples were available for the current analysis. Specific additional permission for the use of these plasma samples in the experiments described was obtained from the Joint Gambia Government/MRC Ethics Committee.

Negative controls for flow cytometry experiments were seven individual sera from UK resident adults who had never been to a malaria endemic area, obtained from whole blood by removal of coagulated cellular material. Each of these donors provided written, informed consent under a protocol approved by the LSHTM Ethics Committee. A pool of these seven negative sera, and three other independent pools of sera from the same source of non-malaria exposed European donors were also used. The serum of an immune Dutch adult whose transmission blocking immunity had been previously assessed as high level using the standardised membrane feeding assay [27] was kindly provided by Teun Bousema, St Radboud University Medical Centre, Nijmegen. This sample was collected more than 10 years prior to the study from an individual who gave verbal, but not written, informed consent to St Radboud UMC for his blood sample to be used for malaria research.

### Definition of transmission blocking from direct membrane-feeding assay (DMFA)

In the DMFA, blood samples were centrifuged, plasma removed and the cells washed as described previously [23], [26]. Cells, including gametocyte-infected erythrocytes, were then resuspended either back in the autologous plasma (AP) sample itself or in control serum (CS) from European donors. This serum had been tested and shown to be permissive for gametocyte survival in *in vitro* cultures. The two parallel preparations of gametocytes were each presented to a cage of approximately 50 adult mosquitoes. Plasma from the Gambian children donating blood for DMFA experiments was classified as having transmission-blocking activity if either of the following two tests were satisfied:

1. transmission occurred among mosquitoes fed with gametocytes in both AP and CS, and the ratio of arithmetic mean oocyst density was significantly higher in the CS cage than in the AP cage [23], [24].
2. transmission occurred only among mosquitoes fed on gametocytes presented in CS, and
  - the proportion of infected mosquitoes in the CS-fed cage was significantly greater than zero by Fisher's exact test, or
  - The proportion of infected mosquitoes in the CS-fed cage was not significantly greater than 0, but at least one mosquito was heavily infected (> = 4 oocysts) in CS and at least 13 uninfected mosquitoes from the AP-fed cage were dissected, or
  - The proportion of infected mosquitoes in the CS-fed cage was not significantly greater than 0, but at least two mosquitoes were infected in CS and at least 13 uninfected mosquitoes from the AP-fed cage were dissected.

Transmission-blocking immunity could not be evaluated in those DMFA experiments in which there were no infected mosquitoes in either cage. A heavy infection was defined as at least 4 oocysts (median of 536 positives among 5000 CS-fed mosquitoes 1998–2002). Thirteen mosquitoes is used as the cut-off because it encompasses the majority of our experiments. The prevalence of infection among 5000 AP-fed insects was 6.5%, and so on average 0.85 out of 13 AP-fed mosquitoes were infected.

### Parasite Culture and Purification

Asexual blood stage parasites were cultured in human type O negative blood and RPMI 1640 (supplemented with 5.96 g l^−1^ HEPES, 2 gL^−1^ sodium bicarbonate, 50 mg l^−1^ hypoxanthine, 3.96 g l^−1^ glucose and 10% blood type AB serum) and incubated at 37°C in 3% CO2/1% O2/96% N2 according to established protocols [28]. Magnet-activated cell sorting (MACS; Miltenyi BioTec, Bergisch Gladbach, Germany) was used to synchronise asexual parasite cultures as follows: at ∼10% mixed stage parasitaemia, the culture was washed three times in PBS supplemented with 2% bovine serum albumin (BSA). The culture was applied to the MACS apparatus using a CS Column mounted with a 21G flow resistor, and washed three times with PBS. Flow-through and washes were subsequently re-applied to the column once. Erythrocytes containing parasites in late developmental stages, and thus with a high content of paramagnetic hemozoin, were retained in the column [29], while non-magnetic early-stage parasites passed through and were collected, washed in PBS/2% BSA, centrifuged at 500*g* for 5 minutes at 25°C and put back into culture with 40 μl/50 ml medium of gentamycin (Gibco, UK). These synchronsied cultures were left to grow for ∼6 hours to ensure viability before gametocyte cultures were established from them.

### Purification of mature gametocytes

Gametocytes were produced as described elsewhere [30], [31] with the following modifications. A magnet-synchronised asexual culture at approximately 6% parasitaemia was used to start 15 ml cultures at a 0.6% parasitaemia and 6% haematocrit. Medium was changed every day until parasitaemia reached 15–20% at approximately day 5, and medium was added to a volume of 25 ml, and thus a haematocrit of 3.6%. N-acetyl glucosamine (NAG) was added at a concentration of 50 mM to kill asexual stages at approximately day 8 [32]–[34] and maintained at this concentration throughout the period of gametocyte culture. Fully mature gametocytes were harvested 17 days after initiation of the asexual culture, and purified by magnetic separation through a MACS column as described previously [35]. Gametocyte maturity was checked by successfully inducing ex-flagellation as described elsewhere [30], [36]. Earlier stages of gametocyte development were synchronized and purified as previously described [37].

### Flow cytometry

The presence of antibodies in plasma recognizing surface antigens on parasite-infected RBCs at different stages of development was demonstrated using an adaptation of a previously established flow cytometry protocol for asexual parasites [35]. In brief, cultures were magnet-enriched and labelled with the nuclear stain ethidium bromide (EB) at 0.1mg/ml per 10^5^ erythrocytes, differentiating infected (nucleated) from non-infected (anucleate) erythrocytes. Aliquots (100 μl) of live, stage-enriched parasite-infected RBCs were incubated for 30 min each with test plasma (5 μl), goat anti-human plasma (diluted 1∶2000) and fluorescein isothiocyanate (FITC)-conjugated rabbit anti-goat immunoglobulin (diluted 1∶25). Samples were washed 3 times between incubations in phosphate buffered saline supplemented with 2% foetal calf serum and kept overnight at 4° C before flow cytometry analysis (FACS Scan, Becton Dickinson). Before each experiment with each of the parasite stages, compensation to exclude background flourescence on the vertical and horizontal axes was carried out on samples single-labelled with EB or FITC, respectively. For gametocyte stages, plasma samples from all 200 Gambian children were tested at the same time, together with control sera. To check that erythrocytes had not ruptured and thus released free gametes, representative samples were examined after flow cytometry to confirm that erythrocyte rupture had not occurred. In addition, side-scatter measurements indicated that host cells remained intact in our flow cytometry experiments. Asexual parasites were tested in 3 separate experiments, with plasma samples batched by year of collection. Data were analyzed using WinMDI software. EB-positive parasite-infected erythrocytes were gated and used to create a 2-dimensional dot blot for each individual sample. Antibody binding was estimated in terms of the percentage cells labelled with both EB and FITC, read in the right upper quadrant (RUQ) of the dot plot. All results shown were confirmed in at least two experiments with the same reagents, but using independent gametocyte preparations. Data analysed are from one of those experiments.

We defined a simple cut-off for positivity. For mature gametocytes, all 200 plasma were run in a single experiment with negative controls in parallel. The mean proportion of events in the RUQ quadrant among the six negative control (unexposed European) plasma was 0.20, with a standard deviation of 0.0305. Plasma were defined as anti-GSA positive sera if with mature gametocytes the proportion of events in the RUQ was above a value of 0.261 (i.e. 26.1% of gametocytes being bound by IgG), representing the mean of the negative controls plus two 2 SD. For asexual parasites, plasma were tested in separate experiments for each year of collection, with negative controls run in each experiment. Mean proportion RUQ for asexual recognition was 0.134, 0.129 and 0.207 for the six negative controls in experiments testing plasma from 2000, 2001 and 2002 respectively. Positivity was then assigned as for gametocytes using the relevant mean plus two SD as cut-off, calculated separately for each of these three experiments.

### Immunofluorescence antibody visualisation of immature gametocytes

Aliquots of early stage gametocytes from the starter flask for subsequent experiments were acetone-fixed on glass slides and stored at −20C. Parasites were counter-stained with ethidium bromide and the incubated with rabbit polyclonal sera recognising Pfs16 (kind gift of David Baker), washed, and incubated with goat anti-rabbit IgG (H _ L), conjugated to AlexaFluor 488 (Cambridge Biosciences). Immunofluorescence was then visualised by confocal microscopy as previously described [34].

### ELISA for antibodies to Pfs48/45 and Pfs 230

The presence of human antibodies of the IgG1 isotype specific for Pfs230 and Pfs48/45 was determined as previously described [38]. Positive and negative controls were included on each plate. A relative recognition ratio was estimated for each plasma for each of the two sexual stage antigens Pfs48/45 & Pfs230 thus: (OD in the ELISA test well–OD in control (no primary antibodies) well)/OD in control well. This was analysed as a continuous variable.

### Data analysis

Statistical analysis was performed using Stata (SataCorp. Stata Statistical Software: Release 8.0, Collage Station, TX: Stata Corporation). Normally distributed continuous variables were compared between categories by Student's t-test; gametocyte densities and oocyst densities were analysed by comparing the ratio of arithmetic means in negative binomial regression as previously described [23], [24]. The χ^2^ (chi) test was used to compare proportions, except where expected values in the 2×2 tables were <6, in which case Fisher's exact test was used.

## Results

### Purity of synchronous gametocyte cultures

**Figure 1:**
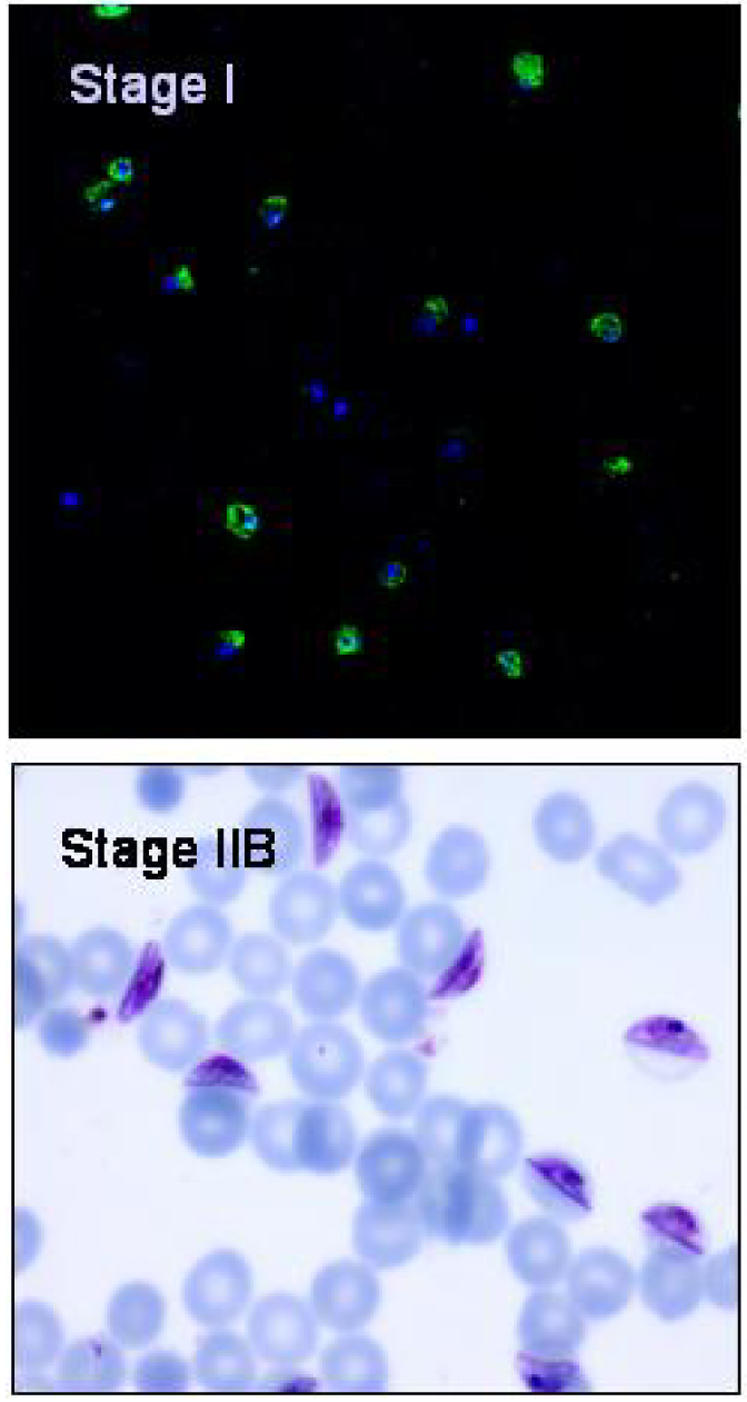
Purification of early gametocyte stages from cultures of 3D7a parasites. The top panel shows stage I gametocytes stained with ethidium bromide (blue) and the sexual parasite-specific anti-Pfs16 antibody (green). The majority of nucleated erythrocytes are also Pfs16 positive and therefore gametocytes. The lower panel shows a Giemsa–stained thin film of the same culture 2 days later, when all parasites have reached Stage IIb of gametocyte development. Asexual parasite contamination was estimated at less than 1%.

Immunofluorescence experiments were performed to verify that asexual parasites had been eliminated from the starting gametocyte culture at day 8, as stage I gametocytes are difficult to discriminate from early trophozoites of the asexual lineage [39]. The majority of ethidium-bromide stained, and therefore nucleated, erythrocytes examined were found to be positively stained with anti-Pfs16 antibody (Fig. 1a). Asexual parasites were estimated to account for fewer than 1% of parasitised cells at this stage. The culture flasks established from these putative gametocyte cultures were examined 2 days later and found to harbour well-synchronised gametocytes at stage IIb (Fig. 1b).

### Recognition of gametocyte-infected erythrocytes by plasma antibodies

**Figure 2:**
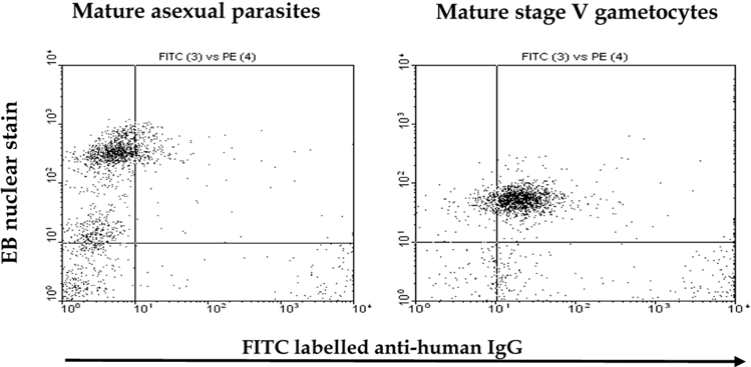
Serum antibodies recognise the surface of mature gametocyte-infected RBCs. Serum from a Dutch individual with previously demonstrated transmission-blocking antibodies was incubated with mature asexual parasites or stage V gametocytes and analysed by flow cytometry. Parasites were dual labeled with FITC conjugate, indirectly recognising human IgG, and EB staining nuclear DNA. Axes denote the number of number of cells counted (events) in each dimension.

Serum from a Dutch adult individual, previously established to have transmission blocking immunity against P. falciparum, was incubated with a culture of mature asexual stages of P. falciparum 3D7a (late stage trophozoites and schizonts) and the proportion of ethidium-bromide positive cells that were recognized by antibodies present in the serum were counted, using flow cytometry. We then performed similar analyses on 4 sequential stages of gametocyte development, each derived from the culture shown in Figure 1. The serum from this individual did not recognize erythrocytes infected with asexual stages, nor those infected with any of the immature gametocyte stages. However, this serum strongly recognised the surface of erythrocytes infected with mature stage V gametocytes (Fig. 2), suggesting the presence of GSA in the form of parasite-encoded molecules, altered host membrane components, or both on the surface of these cells.

We then tested plasma from 200 Gambian children, to investigate further the kinetics and nature of development of the antibodies to surface antigens on RBCs infected with asexual parasites, with developing sexual stages, or with mature gametocytes of *P. falciparum*. These children donated blood for membrane feeding experiments (DMFA) 7, 10 or 14 days after treatment as previously described [23]–[25]. The mean age of the children was 5.0 years (95% C.I. 4.71–5.38), and 49 of them (24.4%) had carried gametocytes at presentation prior to treatment (day 0). Transmission blocking activity was assessed for each of the plasma using results from the DMFA. Mosquito infections occurred in 71 of the 199 experiments in which mosquitoes survived until dissection, 62 of these experiments generated interpretable data in both cages due to adequate mosquito survival and in 18 of these (29.0%) there was evidence of transmission-blocking activity by one of the criteria given in Materials and Methods.

There was no recognition of any immature (stage I–IV) 3D7 gametocytes among the 194 plasma for which data were obtained (not shown). Sixty-six (34.0%) of these plasma recognized GSA on the surface of RBCs infected with mature stage V gametocytes, with a mean proportion of 39.5% of cells in the RUQ (inter-quartile range 33.0 to 44.6% ; positivity cut-off was 26.1%). Examination of material after flow cytometry confirmed that gametocytes remained inside intact host erythrocytes, and no appreciable gamete release had occurred during these experiments.

**Figure 3:**
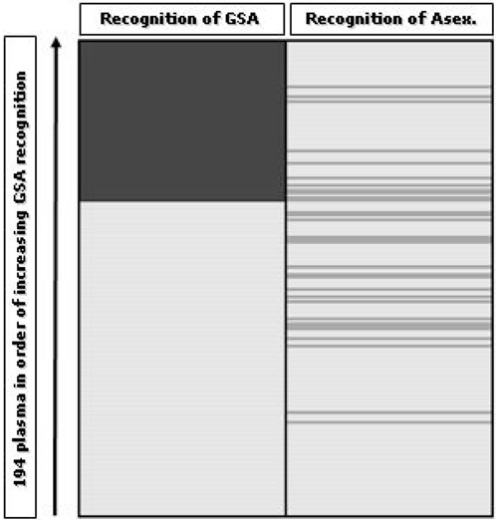
Recognition profiles of plasma IgG from 202 Gambian children. Erythrocytes harbouring P. falciparum clone 3D7a stage V gametocytes (left column) and asexual parasite stages (right) were tested with each of 194 plasma (rows). Plasma are arranged in increasing order of the proportion of gametocyte recognition events in the right upper quadrant of the flow cytometry dot-blot. Positive antibody recognition is scored as dark grey. Pale fill indicates that antibodies could not be detected above the level of controls (see text).

Fifty-three (26.4%) of the 200 plasma successfully tested recognised the surface of erythrocytes infected with asexual parasites. Eleven test sera recognized both GSA and the surface of asexual-infected erythrocytes, but there was no association between these two reactivities. The relationship between recognition of sexual and asexual parasites is shown for each plasma in Figure 3 and Table 1. This suggests these two parasite life cycle stages place distinct antigen sets on the host erythrocyte surface. Further, anti-GSA antibodies were more common among older children (mean age difference 0.621 years, 95% C.I. 0.041–1.283 years; P = 0.066), but this was not true of antibodies against the 3D7 asexual parasite surface (mean age difference 0.349 years, 95% C.I. −0.516–1.22 years; P = 0.787).

**Table 1:** Associations between recognition of asexual- and sexual-stage parasites in flow cytometry, carriage of gametocytes at presentation and transmission-blocking immunity on plasma from 200 treated Gambian children.

Odds Ratio, (95% C.I.)	Antibodies to Mature gametocytes Stage V	Antibodies to Mature Asexual Stages
*Antibodies to Asexual stages*	1.14, (0.454–2.73) P = 0.756	∼
*Gametocyte carriage at presentation*	0.531, (0.225–1.18) P = 0.098	1.38, (0.513–3.44) P = 0.461
*Transmission blocking immunity * *	1.59, (0.398–5.89) P = 0.435	5.85, (1.28–27.4) **P = 0.010**

* Only data from feeding experiments with at least one positive mosquito are included (N = 71).

**Table 2:** Associations between relative recognition of Pfs48/45 sand Pfs230 antigens, and the presence of antibodies against either GSA or asexual parasite surface antigens, carriage of gametocytes at presentation and transmission-blocking immunity in 65 plasma from Gambian children.

Mean difference in relative recognition (95% CI)*	Antibodies to Pfs48/45	Antibodies to Pfs230
*Antibodies to Mature gametocytes (Stage V)*	0.533 (−0.780–1.85) P = 0.420	1.47 (0.429–2.51) **P = 0.006**
*Antibodies to Asexual stages*	0.875 (−0.059–1.81) P = 0.066	1.18 (0.433–1.93) **P = 0.003**
*Gametocyte carriage at presentation*	0.740 (−0.171–1.65) P = 0.110	0.754 (−0.006–1.51) **P = 0.052**
*Transmission blocking activity * **	0.56 (−0.757–1.88) P = 0.393	1.47 (0.364–2.58) **P = 0.011**

* Relative recognition is a ratio, and has no units (see Materials & Methods); mean difference can be positive or negative. If the 95% CI is wholly positive, then the patient plasma indicated had a significantly higher mean fluorescence in the ELISA, whereas a wholly negative 95% CI would indicate significantly lower mean fluorescence in the ELISA. P value from 2-sided t-test of Student.

** Data from samples collected in 2000 and infecting at least one mosquito are included (N = 35; see Table 1).

Surprisingly, day 7, 10 or 14 plasma antibodies that recognised GSA were less common among children that had harboured gametocytes prior to treatment, although this was not statistically significant (Table 1). Children with plasma antibodies that recognised GSA in the flow cytometry assay were no more likely to exhibit transmission-blocking activity in the DMFA than other children. However, transmission blocking activity was significantly more common among children with antibodies recognising asexual parasites (Table 1), suggesting this may be related to either the longevity of infection, or the parasite load, but we did not have sufficient data from the DMFA to distinguish these possibilities. Mild anaemia, which is an indicator of a malaria infection of long duration, was unrelated to the presence of antibodies recognising either GSA or the surface of asexual parasites (data not shown).

To further test whether the development of anti-GSA and anti-asexual antibodies depended on duration of infection, we compared the prevalence of antibodies in plasma taken at day 7 (N = 176) and at days 10 or 14 (N = 21). GSA antibodies were found in 11 of 20 samples from day 10 or 14 that were tested (55.0%) and only among 55 of 171 samples taken at day 7 (32.2%; O.R. 2.58, 95% C.I. 0.907–7.45; P = 0.042). Antibodies recognising asexual parasites did not occur in any of the plasma samples taken at day 10 or 14, but were present in 31 of 174 samples taken at day 7 (17.8%). Thus antibody reactivities to GSA, but not the surface of 3D7 asexual parasites, were more common in plasma taken 10 days or more after treatment than they were in day 7 plasma.

### Antibodies to GSA and recognition of gamete antigens Pfs48/45 and Pfs 230

Gamete antigen ELISA assays were conducted for each of 65 plasma collected in 2000. The relative Pfs48/45 and Pfs230 recognition score of each was tested for association with transmission-blocking immunity as measured by DMFA, and with the ability to recognise both GSA and the surface of asexual parasites. Results of these analyses are presented in Table 2. Plasma samples with antibodies that recognised gametocytes, and those that recognised asexual parasites exhibited significantly stronger recognition of Pfs230 (but not Pfs48/45) in ELISA. The same was true of plasma with demonstrable transmission-blocking activity.

### Antibodies to GSA and persisting carriage of gametocytes

To explore the possibility that antibodies detected in the day 7 plasma of children carrying gametocytes may have affected gametocyte survival in those hosts, we examined subsequent gametocyte carriage in children with and without plasma antibodies to GSA. Children carrying anti-GSA plasma antibodies at the day blood was donated for DMFA were found to have a lower mean gametocyte density at day 28, but this was affected by the treatment received. Among children followed up to day 28 and receiving non-artemisinin treatments (CQ, SP or CQ/SP; N = 99), the presence of anti-GSA plasma IgG was associated with significantly lower gametocyte densities at day 28 (ratio of means 0.139, 95% C.I. 0.037–0.526; P = 0.004). This was not seen among the 67 children contributing to this analysis who received CQ/AS or AL (ratio of means 0.643, 95% C.I. 0.008–50.5; P = 0.84). Thus, in our studies at least, artemisinin treatment is a more important determinant of gametocyte persistence than the presence of anti-GSA antibodies. Gametocyte densities at day 28 were significantly higher among children receiving CQ, SP or CQ/SP with anti-asexual parasite antibodies present in the blood sample donated for DMFA (ratio of means 29.7 , 95% C.I. 5.08–174; P <0.001; N = 101), whereas this was not the case in children treated with ACT.

## Discussion

In this study of malaria patients identified as gametocyte carriers, we have demonstrated that erythrocytes infected with mature gametocytes of *P. falciparum* carry surface antigens that are recognised by naturally-elicited antibody responses. Our results provide four lines of evidence that these GSA are distinct from surface antigens expressed by the asexual stages of cultured 3D7 parasites. Firstly, serum from a Dutch adult with a previously demonstrated high titre of transmission-blocking antibodies (T. Bousema, P. Schneider and R. Sauerwein, personal communication) strongly recognised the surface of erythrocytes infected with stage V gametocytes, but did not recognise the surface of erythrocytes infected with 3D7 asexual parasites (Fig. 2). Secondly, among the 200 plasma from Gambian donor children, carriage of IgG that recognised GSA was not associated with recognition of the surface of erythrocytes harbouring 3D7 asexual parasites. Thirdly, anti-GSA antibodies were significantly more common in plasma samples taken on days 10 or 14 after treatment, compared to day 7 plasma, whereas anti-asexual parasite antibodies were found only in day 7 samples. Finally, among children treated with non-artemisinin therapy, the presence of anti-GSA antibodies in day 7, 10 or 14 plasma was significantly associated with lower gametocyte densities at day 28, whereas anti-asexual parasite antibodies were associated with significantly higher gametocyte densities at day 28.

These observations need to be interpreted in the light of the unique dynamics of gametocyte emergence in *P. falciparum* infections, where in successfully treated individuals there is a delayed peak of peripheral gametocytaemia approximately 7 days after clearance of asexual parasites[40], whereas the infectiousness of circulating gametocytes to mosquitoes is highest at 10–14 days [25]. Where patent or sub-patent persistence of asexual parasites occurs, gametocyte carriage is significantly extended [41], and therefore the length of time an individual is exposed to viable circulating gametocytes, and thus likely to acquire antibodies recognising GSA, will differ according to treatment outcome, and to the intrinsic anti-gametocyte effects of the treatment received.

Plasma antibodies that block transmission of *P. falciparum* and *P. vivax* to mosquitoes were first described several decades ago, and are currently of interest for the development of transmission-blocking vaccines that target gamete antigens [42]. We measured transmission-blocking activity in our series of Gambian blood donors by DMFA [23], and found no association with recognition of GSA. However, the DMFA has several drawbacks for identification of transmission-blocking activity compared to the standardised membrane feeding assay, SMFA, [27], most notably that blood-fed mosquito cages in which no transmission occur are not informative. Therefore it would be useful to have our panel of plasma also tested in the SMFA.

One of the unexpected findings of our study is that none of the sera or plasma tested recognised the surface of erythrocytes harbouring immature stages of 3D7 gametocytes. Serum antibodies from a Dutch adult with known transmission-blocking immunity were found to recognise the surface of erythrocytes infected with Stage V gametocytes that were demonstrably competent for exflagellation , but not of those infected with immature developing gametocytes. Similarly, none of the Gambian plasma recognised immature gametocytes. Therefore it appears that the GSA recognised by the antibodies we have described are distinct from the putative ligands responsible for sequestration of immature gametocytes during their development [20], [34]. Possible explanations for this apparent contradiction include, firstly, that the adhesins of immature gametocytes are highly polymorphic and strain-specific, and those of clone 3D7 were not recognised by immune sera from The Gambia. Secondly, it may be that gametocyte adhesins are cryptic or non-immunogenic molecules, or there is a deficiency in the 3D7 clone that prevents the normal expression of gametocyte adhesins *in vitro*. Finally it remains possible that sequestration of immature gametocytes *in vivo* does not require the expression of adhesins on the erythrocyte surface. Experiments with gametocytes derived from parasites from a variety of genetic backgrounds are needed to test these possibilities, and to determine whether our observations made with the 3D7 clone are generally applicable.

The identity of the GSA we have described remains unknown, and we cannot dismiss the possibility that altered host membrane proteins are targets of the recognition we have observed. There is an association between parasite-encoded STEVOR proteins and the erythrocyte membrane in late stage cultured gametocytes of the 3D7 line [34], and a subset of these, or of the related rifin family may be recognised by anti-GSA antibodies. Other possible protein targets of anti-GSA antibodies include members of the PfEMP1 family, as a subset of *var* genes are known to be transcribed in gametocytes [37], and the LCCL-domain protein pSLAP, which is associated with the plasma membrane of erythrocytes infected with late stage *P. falciparum* gametocytes [43]. Future work will employ recombinant forms of candidate GSA, and specific antibodies raised against these reagents, to identify the key molecules involved in this natural immune response to the transmissible form of *P. falciparum*.

The identity of the antigens recognised on the surface of erythrocytes infected with asexual parasites in our experiments is also unclear. In the absence of phenotypic selection using anti-VSA antibodies or adhesion panning, 3D7 cultures express a surface phenotype characterised by relatively low level CD36- dependent endothelial adhesion, and these parasites are poorly recognised by plasma antibodies from semi-immune children [44]. This explains the lack of association between the age of our plasma donors and recognition of the surface of erythrocytes infected with asexual parasites. Therefore it is possible that asexual parasites with a surface antigen phenotype dominated by the highly recognised Type A var genes, such as the antibody-selcted 3D7-Dodowa1 line [11], may have been more commonly recognised by plasma antibodies from children who recognised GSA. What we can say with confidence is that mature 3D7 gametocytes in our synchronised cultures expressed molecules on the host erythrocyte surface that were not found on erythrocytes harbouring 3D7 asexual parasites from the same cultures.

A subset of our test plasma (those collected in 2000) were analysed for recognition of recombinant forms of the gamete antigens Pfs48/45 and Pfs230. Recognition of the latter, but not the former, was significantly associated with antibodies recognising both GSA and asexual antigens in the flow cytometry assay, and with transmission-blocking activity in the DFMA. This is partially consistent with previous work, which has shown an association between the presence of antibodies recognising these two gamete antigens with transmission-blocking activity in the SMFA [14], [15]. In most studies, prevalence of antibodies to Pfs230 increases with age and exposure [38], but it is unknown whether acquisition of antibodies to GSA may follow a similar pattern.

An exploratory analysis suggests that the presence of anti-GSA antibodies 1 to 2 weeks after treatment of clinical malaria with non-ACT regimens are associated with lower gametocyte densities 4 weeks after treatment. Interestingly, anti-asexual parasite antibodies were associated with higher day 28 gametocyte densities in this same group of treated children, suggesting that in these individuals sub-patent asexual parasitaemia may be persisting, giving rise to both antibody responses and subsequent gametocytaemia [41]. The role of anti-GSA antibodies in controlling gametocytaemia *in vivo* needs to be further investigated in longitudinal studies of gametocyte carriage, but it does appear from results presented here that such an effect may only be significant in the absence of ACT treatment. Further longitudinal studies and cross-sectional serological surveys of anti-GSA antibodies in children with and without gametocytaemia are needed to further test the association between these antibodies and gametocyte carriage.

Our results present the first, albeit partial, picture of the immune response to GSA. The antibodies that recognise GSA are more common in older children, and are distinct from immune responses directed against asexual parasite surface antigens in our unselected cultures. Further, parasitological follow-up data suggest anti-GSA antibodies are associated with ability to control gametocyte densities *in vivo*. We propose that the generation of vaccine-elicited immunity targeting GSA may represent a feasible new strategy aimed at blocking the transmission of *P. falciparum* in endemic settings, and thus further epidemiological and biological studies of anti-GSA responses are needed.
